# The luminance-response function of the photopic negative response (PhNR): analysing different stimulation, recording and measurement approaches

**DOI:** 10.1007/s10633-026-10102-0

**Published:** 2026-04-07

**Authors:** Oliver R. Marmoy, Emma Hodson-Tole, Dorothy A. Thompson

**Affiliations:** 1 Clinical and Academic Department of Ophthalmology, Great Ormond Street Hospital for Children, London, UK; 2 UCL-GOS Institute of Child Health, London, UK; 3 Department of Life Sciences, Manchester Metropolitan University, Manchester, UK; 4 Institute of Sport, Manchester Metropolitan University, Manchester, UK

**Keywords:** Electroretinogram, PhNR, Electrode, ERG, Photopic negative response, Retinal ganglion cell

## Abstract

**Purpose:**

The photopic negative response (PhNR) is a measure of generalised retinal ganglion cell function. There has been heterogenous methodology to record this, with varied electrode type, stimulus luminance, temporal frequency and measurement approach. This study aimed to empirically explore these features to identify an optimal PhNR luminance-response protocol which produces the lowest variance and maximal efficiency to guide clinical protocols.

**Methods:**

Twelve healthy participants were recruited (age range 27-51y). Flash ERGs were simultaneously recorded from infraorbital skin and corneal fibre electrodes to a range of red flash stimuli (− 0.3–2.4 log cd.s/m^2^, incremented in 0.3 log units), whilst varying temporal frequency (1–5 Hz), background blue luminance (1, 1.5, 2 log cd/m^2^), and PhNR measurement approach (from baseline or b-wave, as an amplitude or ratio). The luminance-response series data were analysed for changes according to these variables, alongside a calculation of variability.

**Results:**

The PhNR luminance-response curves showed few significant differences with increasing temporal frequency, though inter-subject variability was highest for the slowest (1 Hz) and highest flash (5 Hz) stimulation rates. Background luminance reduced the relative sensitivity (*K*) but not maximal amplitude of the luminance-response curves (V_max_). With skin electrodes the b-PhNR amplitude and b-PhNR ratio showed the lowest levels of variability compared with other measurement approaches or electrodes.

**Conclusion:**

This study demonstrates that temporal frequency can be increased significantly, optimally at 4 Hz, without compromising the PhNR. PhNR variance is lower with skin electrode recordings and PhNR amplitude measurements from the b-wave compared to corneal fibre electrodes and baseline-PhNR amplitudes.

**Supplementary Information:**

The online version contains supplementary material available at 10.1007/s10633-026-10102-0.

## Introduction

The photopic negative response (PhNR) is a slow negative potential that follows the b-wave of the light adapted full-field ERG. The PhNR arises predominantly from spiking retinal ganglion cell (RGC) activity [1], though it likely has contributions from other proximal retinal cells, particularly those with inward rectifying K^+^ channels such as amacrine or Müller cells [2–5]. The clinical applications of the PhNR are therefore wide. Although the PhNR may be used to assess generalised RGC function [6], it may be less specifically applied in other diseases affecting other retinal cells such as the inwardly rectifying K^+^ channels [5].

The International Society for Clinical Electrophysiology of Vision (ISCEV) extended protocol describes a PhNR produced to a single flash strength at 1 Hz [7]. This allows the PhNR to recover to baseline but is time consuming. A range of flash strengths is needed to capture the PhNR sensitivity akin to the luminance response function of the photopic b-wave [8, 9], which has been modelled successfully as a combination of Gaussian and logarithmic growth functions [10]. The effect of increasing flash luminance on the PhNR is less well characterised, but is important because it may provide better evaluation of RGC function and sensitivity changes.

**Fig. 1:**
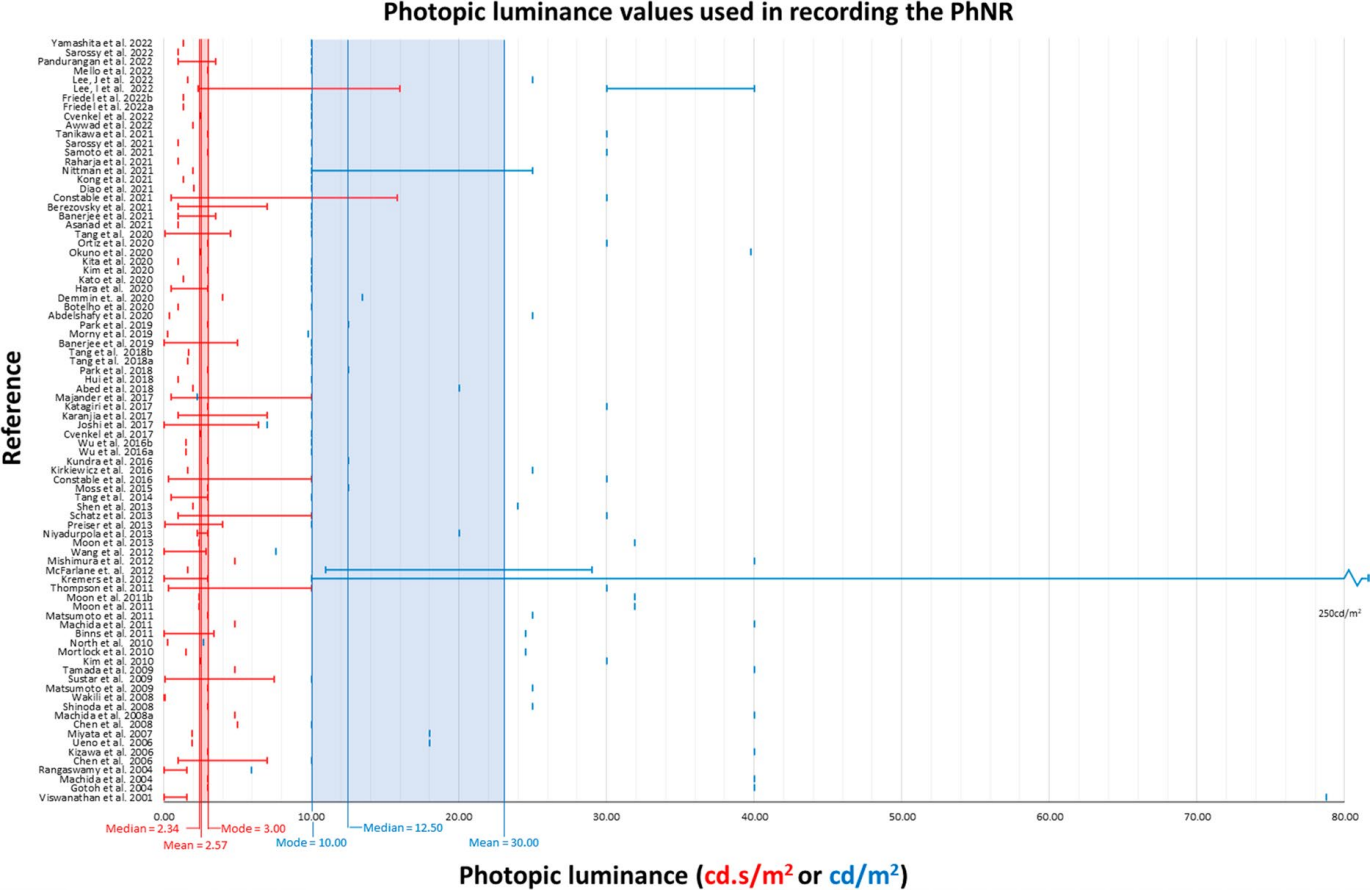
Meta-analyses of existing studies incorporating the PhNR in human participants (from 1998 to 2022). These data include only those studies specifying luminance stimulation parameters. Troland values were converted to candela using mean or presumed pupil diameters provided in the study of interest (or 7 mm diameter for those using RETeval stimuli) to calculate the pupil area, then candela equivalent calculated by dividing the troland value by pupil area. The cd/m2 to cd.s/m2 conversion was performed by dividing the value by 1000 and multiplying by stimulus duration in milliseconds. Scotopic to photopic conversion was performed by calculating the relative scotopic and photopic sensitivity at a specified wavelength according to the photopic and scotopic Vλ functions (CIE 1978 and 1951, respectively). Importantly, these data included a range of stimulus chromaticity

A meta-analysis of the published PhNR studies in human participants (from 1998 to 2022) highlights the wide range of stimulus and background luminance values used to maximise PhNR amplitude [11]. The most common, averaged flash stimulus luminance is 2.6 cd.s/m^2^ (median 2.3 cd.s/m^2^) (Fig. 1). Background luminance also varies in relative luminance, which adjusted for chromaticity is on average 30 cd/m^2^ (median 12.50 cd/m^2^) (Fig. 1).

The PhNR amplitude increases with increasing flash luminance to a maximum (*V*_max_) around 1.5–2 photopic trolands/sec [12–17]. This ascending limb of PhNR amplitude increase can be modelled with the Naka-Rushton function [16]. It has been suggested that the PhNR to strong flash strengths shows a decrease in amplitude, but few have explored flash luminance beyond *V*_max_ to characterise or model this apparent PhNR ‘hill’ [17–19].

This study aimed to assess the luminance-response function of the PhNR and how this is affected by temporal frequency, electrode type and background luminance. The effect of these factors on PhNR variability were studied empirically to support more efficient recording of the PhNR luminance-response function, to help develop more time efficient PhNR recording protocols for clinical practice.

## Materials and methods

### Ethics

This project was given favourable ethical opinion by the NHS Health Research Authority (ref. 20/SC/0345) alongside university research ethics and governance approval (ref. 24880). Written informed consent was obtained from all study participants. All study activities conformed to the most recent revision of the Declaration of Helsinki (2013). This study did not use any animal subjects. The authors declare no conflicts of interest.

### Participants

Twelve healthy participants (8 females, 4 males) were recruited (mean 33.5 years, range 27–51). Participants denied history of ophthalmic or neurological conditions apart from mild refractive error (< 6.00DS). Heidelberg SPECTRALIS (Heidelberg Engineering Ltd., UK) Optical Coherence Tomography (OCT) measures of peripapilliary retinal nerve fibre layer (RNFL) thickness, posterior pole ganglion cell layer volume (GCCV) and Bruch’s membrane opening (BMO) minimum rim width values were within manufacturer provided reference ranges for all participants. Ten participants were Caucasian and two participants were Asian (both male). Eye colour was recorded (blue = 2, blue-green/hazel = 6, brown = 2).

### ERG recording

ERGs were recorded simultaneously by two electrode types; (1) skin electrodes using self-adhesive infraorbital surface electrodes (Natus®, Disposable Adhesive Electrodes), (2) corneal fibre silver thread electrodes (Spes Medica, Genova, Italy). Both types of electrodes were referred to the ipsilateral lateral outer canthus with a ground electrode placed on the forehead. Impedances were below 5 kΩ and balanced within 20%.

Data were acquired using an Espion E3 electrophysiology system (Diagnosys Vision Ltd., Dublin) with signals sampled at 1 kHz and band-pass filters set to 0.3–300.3 Hz. A pre-stimulus baseline was maintained at 5 ms. An automated artefact rejection was enabled for signals exceeding 200 µV. The post-stimulus epoch was altered according to the temporal frequency of stimuli, resulting in epochs of 250 ms, 200 ms, 200 ms, 150 ms and 120 ms for 1, 2, 3, 4 and 5 Hz temporal frequencies, respectively. ERGs were recorded binocularly. A linear detrend or manual removal of individual traces was performed data which appeared to be obviously confounded by blink, eye movement or other artefacts. A minimum of 100 trials were obtained per average, with a minimum of two averages overlaid to demonstrate repeatability.

### Flash stimuli

Flash stimuli were delivered using a hand-held LED flash stimulator (ColorFlash™, Diagnosys Vision Ltd., Dublin) to natural pupils, similar to that used in alternative paediatric ERG protocols [20]. The device was positioned ~ 6.5 cm away from the bridge of the nose meaning the circular flash aperture subtended a horizontal visual angle of ~ 92 degrees (6,648 deg^2^ field). Participants were instructed to look between the center and bottom of the flash aperture, placing the eyes slightly in downgaze. Red flashes (λmax = 630 nm) were superimposed on a steady blue (λmax = 455 nm) background. Red flash strengths were presented in ascending 0.3 log increments between − 0.3 and + 2.4 log photopic cd.s/m^2^. Flash repetition rate ranged between 1 and 5 Hz (1 Hz increments; 1–5 flashes per second) with constant background luminance of 10 cd/m^2^ (1 log unit). Background luminance was independently studied by presenting 3 Hz flashes on two additional blue backgrounds and allowing five minutes adaptation before recording; 30 cd/m^2^ and 90 cd/m^2^ corresponding to ~ 1.5 (1.48) and ~ 2 (1.95) log cd/m^2^ respectively. The recording order was randomised [Microsoft excel simple randomisation function] to minimise order effects.

### Signal measurement and analysis

**Fig. 2:**
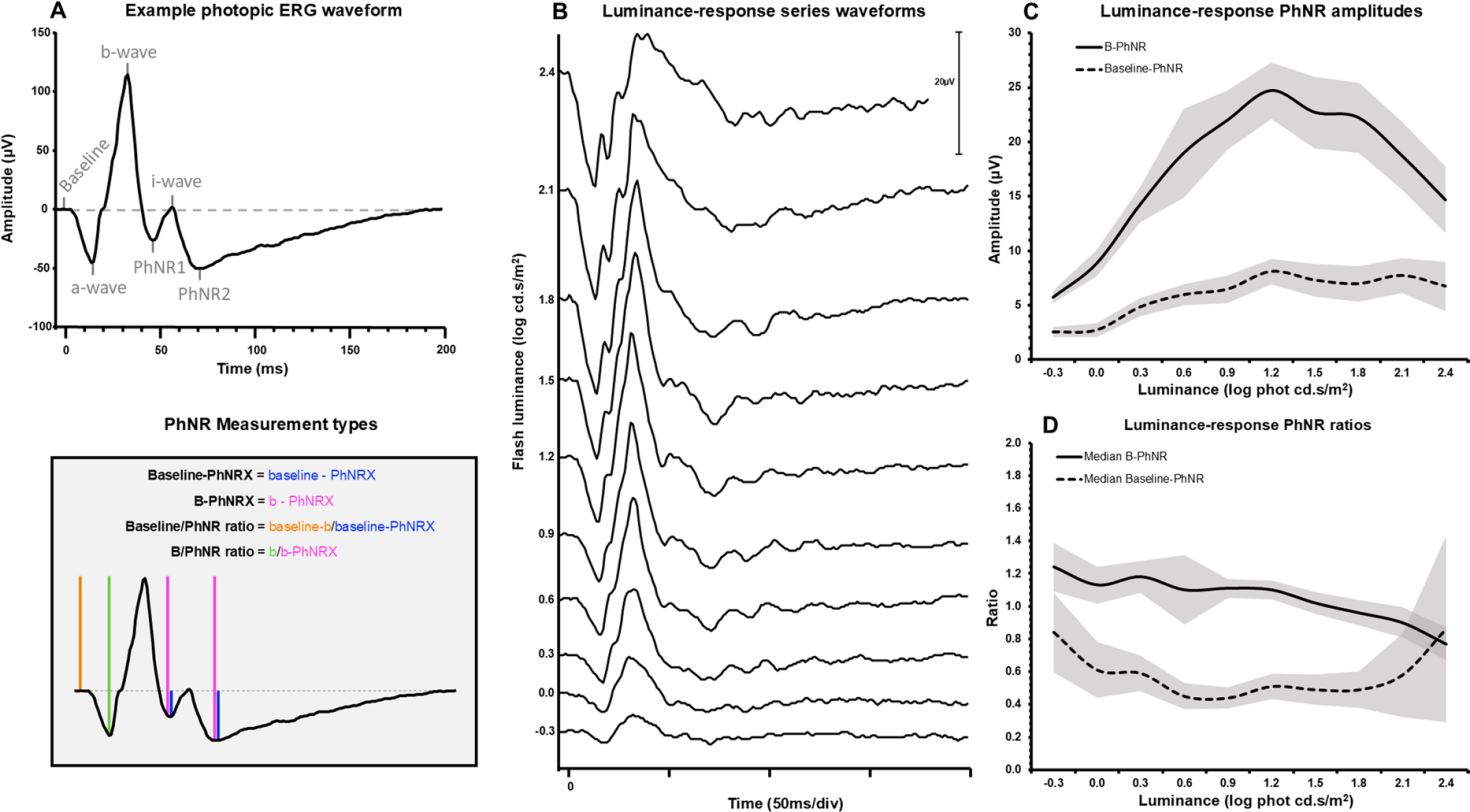
Light adapted electroretinogram showing photopic negative response measurements (**A**) and illustrative luminance-response series (**B**) with plotted amplitudes and ratios (**C–D**). **A**: An illustrative waveform is seen with the a-wave, b-wave, PhNR1, i-wave and PhNR2 labelled. The measurement approaches used in this study are illustrated in the shaded box and corresponding coloured lines represent each measurement. PhNRX signifies PhNR1 or PhNR2, dependent on analysis. **B**: an example of individual participant data, with panels C and D exhibiting group data from PhNR2 (n = 12, 24 eyes) for the skin electrode, all from the 3 Hz temporal frequency protocol. Panel **C** plots the median B-PhNR amplitude (solid line) and median baseline-PhNR amplitude (dashed lines) ± 95% confidence intervals across increasing flash luminance. Panel **D** plots the median B-PhNR ratio (solid line) and median baseline-PhNR ratio (dashed line) ± 95% confidence intervals across increasing flash luminance

The signals were measured by an experienced electrophysiologist at the authors’ institution and verified by the lead investigator (ORM). The ERG measurements are described in Fig. 2. At the strongest flash luminance the true peak of the b-wave measurement was sometimes ambiguous due to waveform obscuration by prominent oscillatory potentials. In such cases, the largest positive deflection was chosen as the b-wave for both amplitude and peak-time.

The PhNR was measured through four different methods to explore the characteristics and variability of each measurement type:

(i) Absolute amplitude from baseline to PhNR (baseline-PhNR)
(ii) Absolute amplitude from b-wave peak to PhNR (b-PhNR)
(iii) As the ratio of amplitudes ‘from baseline to b-wave’/baseline to PhNR (i) (baseline/PhNR ratio)
(iv) As the ratio of a-b amplitude/amplitude of b-PhNR (ii) (b/PhNR ratio).

The above measurements focused upon the PhNR at its major negative trough, typically between 65 and 85 ms following the i-wave (termed PhNR2), but the negativity before the i-wave (termed PhNR1) was also measured as this has been used in a small number of previous studies [5, 21–23]. At the extremes of low and high flash luminance levels, the i-wave was sometimes indistinct or exhibited slow later oscillations [24]. In these circumstances, the luminance-response waveform series was visualised to guide measurement position of the PhNR1.

A Gaussian model was created and applied to each participant’s data using the ‘NonlinearModelFit’ function in Wolfram Mathematica (version 11.1.1). This provided a means to explore the wider luminance-response series of the PhNR by extracting coefficients *V*_max_, *K*, *µ* and *σ*, which are described below. The Gaussian was defined as:

$$V_{PhNR} \left( L \right) = C \cdot e^{{ - \frac{1}{2}\left( {\frac{L - \mu }{\sigma }} \right)^{2} }} ,$$

Where *µ* represents the log luminance (*L*, in log cd.s/m^2^) at which the peak PhNR (*V*_max_) occurs, *σ* the standard deviation (i.e. width or spread of the curve), and *C* is a composite scaling component, $C=\frac{A}{\sigma \sqrt{2\uppi }}$, where *A* determines the overall amplitude of the response. Finally, the semi-saturation constant *K* was calculated as the lower flash luminance at which 0.5 *V*_max_ occurs (in log cd.s/m^2^). The model fit was assessed using the root-mean square error (RMSE). A Gaussian model presumes a symmetrical curve of the PhNR luminance-response series, however it is known that the PhNR luminance-response can be variably asymmetric [19, 25]. Preliminary analysis showed some individuals exhibited a plateau in amplitude to the strongest flashes, therefore the Gaussian model was run for the full data, and again minus one then two data points from the strongest flashes. The model which produced the lowest RMSE for that individual’s data series was selected for analysis (Supplemental Table 1, illustrated in Supplemental Fig. 1) to account for this variable asymmetry seen in some individuals. Visual observation of model fits against data points showed that a RMSE ≥ 20 was associated with a poorer model fit to the data, therefore values exceeding this were excluded from analysis.

**Table 1:** Statistical significance (* = *p* < 0.05 following Bonferroni correction) for PhNR measurement type with altering temporal frequency, according to each flash luminance strength

		PhNR differences with temporal frequency Flash luminance (log cd.s/m^2^)									
		– 0.3	0	0.3	0.6	0.9	1.2	1.5	1.8	2.1	2.4
Corneal fibre electrode	Baseline-PhNR amplitude	*	–	*	*	*	*	–	–	–	–
	B-PhNR amplitude	–	–	–	–	–	–	–	–	–	–
	Baseline-PhNR ratio	*	––	*	*	*	*	*	-	–	–
	B-PhNR ratio	*	–	*	*	*	–	–	–	–	–
Skin electrode	Baseline–PhNR amplitude	–	–	*	*	*	*	*	*	*	*
	B-PhNR amplitude	–	–	–	–	–	–	–	–	–	–
	Baseline-PhNR ratio	–	–	*	*	*	*	*	*	*	*
	B-PhNR ratio	–	–	*	*	*	*	*	*	–	*

### Statistical analysis

Data for the a-wave, b-wave and PhNR were analysed in IBM SPSS statistics (v2.0.1.1) and Microsoft Excel (v2301). Each eye was included independently but each variable was assessed through paired tests to account for the lack of independent change. Kruskal–Wallis tests were used to assess whether PhNR measurements at each luminance point across the luminance-response series differed with stimulus (temporal frequency, background luminance) and recording (electrode type, PhNR measurement) variables. This was also performed for a- and b-wave amplitudes across stimulus and recording variables. All statistical *p*-values were those subject to a Bonferroni correction by multiplication of the *p*-value by number of comparisons made, with those *p*-values stated are those following correction, which was completed to reduce risk of type-1 error given the multiple comparisons made.

The major PhNR model parameters (*V*_max_, *K, σ, µ*), provided by the Gaussian fit, were assessed for normality using a Shapiro–Wilk test with Kruskal–Wallis comparative statistics used to compare model parameters derived from the different stimulus characteristics.

The variance in PhNR data were calculated using the coefficient of quartile deviation (CQD), described by the difference between upper and lower quartiles (Q) by the following formula (Q_3_–Q_1_)/(Q_3_ + Q_1_). The CQD was calculated to study the differences in variance according to PhNR electrode type, flash variable and measurement approach.

## Results

The Gaussian model fitting parameters and subsequent RMSE are provided in Supplementary Table 1.

### PhNR alterations with temporal frequency

**Fig. 3:**
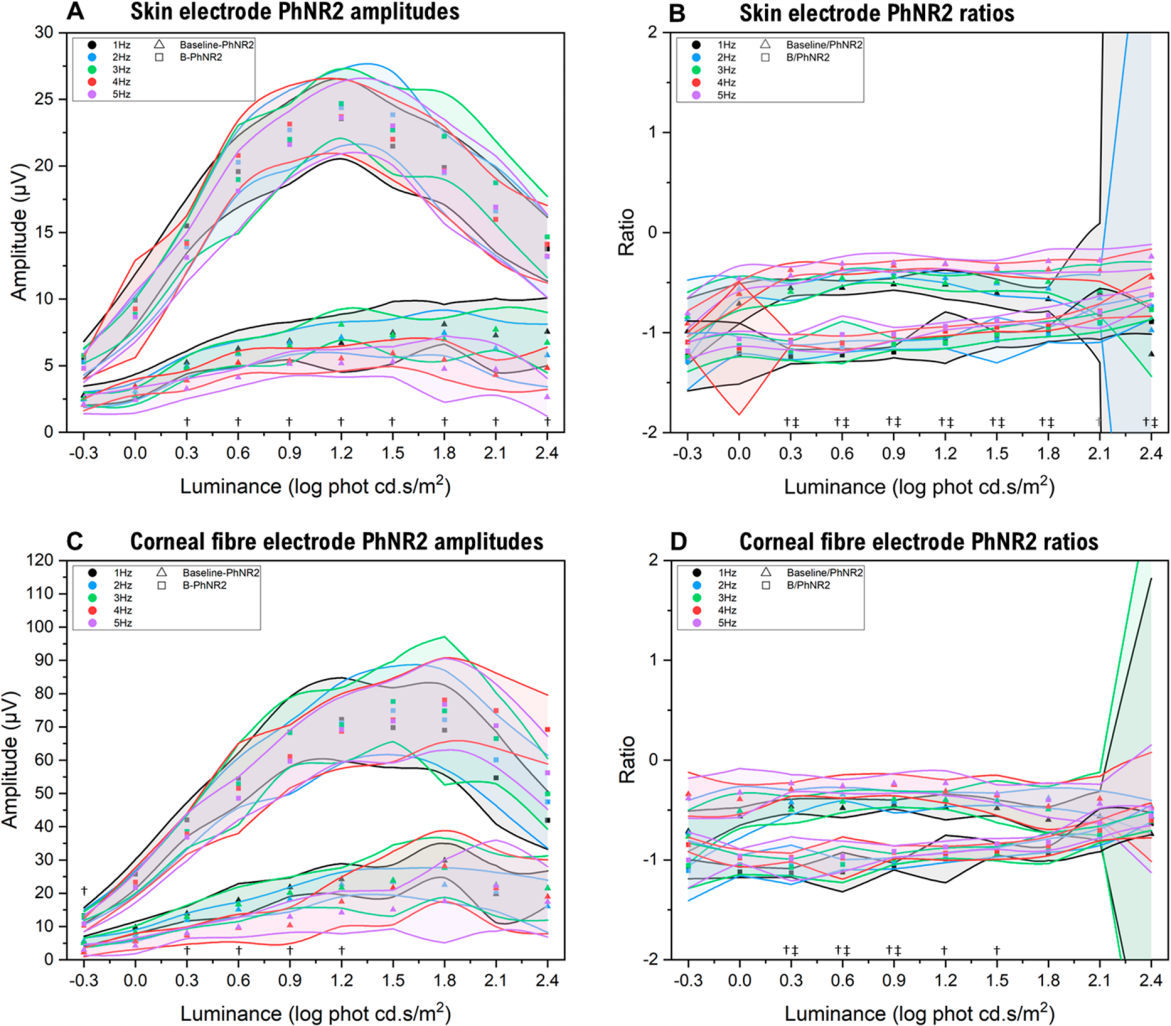
Effect of temporal frequency on the luminance-response function of the PhNR. For all panels, the median baseline-PhNR measurements are indicated by triangles, with B-PhNR measurements indicated by squares. The shaded areas around the median points are the 95% confidence intervals for each data point. Panel A shows the absolute baseline-PhNR2 and B-PhNR2 amplitudes for the skin electrode across different temporal frequencies (1–5 Hz). Panel B shows the baseline/PhNR2 ratio and B/PhNR2 ratio for the skin electrode across different temporal frequencies. Panel C shows the absolute baseline-PhNR2 and B-PhNR2 amplitudes for the corneal fibre electrode across different temporal frequencies (1–5 Hz). Panel D shows the baseline/PhNR2 ratio and B/PhNR2 ratio for the corneal fibre electrode across different temporal frequencies. Significant differences are indicated by † = significance observed for baseline-PhNR measurements at that flash luminance, and ‡ = statistical significance observed for B-PhNR measurements at that flash luminance

The effects of temporal frequency on the absolute amplitude of B-PhNR and baseline-PhNR were first assessed at each luminance point (− 0.3 to 2.4 log cd.s/m^2^) for the grouped data (Fig. 3). Then, each subjects *V*_max_ and *K,* derived from the Gaussian model parameters were assessed at each temporal frequency. PhNR1 is seldom used but the effects of temporal frequency are provided in Supplemental Fig. 2.

A summary of statistical significance, following correction for multiple comparisons, is shown in Table 1 for changes in absolute amplitude and ratio with temporal frequency and measurement approach. Although statistical significance was revealed through these comparisons, the relative effect size appeared small (Fig. 3) and appeared minimal when modelled with the Gaussian curve.

### Absolute amplitude

Absolute amplitudes measured from the baseline showed significant differences with increased temporal frequency for both electrode types. For the corneal fibre electrode, significant differences were observed at lower flash luminance for the baseline-PhNR2 amplitudes, tending to smaller amplitude as temporal frequency increased. This was more widespread across flash luminance range for the skin electrode baseline-PhNR2 amplitude (Fig. 3 and Table 1). Conversely, no significant differences were observed in the B-PhNR2 amplitude and altered temporal frequency, for either electrode type.

### Amplitude ratio

The baseline-PhNR ratios showed similar trends whereby temporal frequency demonstrated significant differences to a range of low-moderate flash luminance for the corneal electrode, which was more widespread across the luminance range for the skin electrode (Fig. 3). The B-PhNR ratio differed from absolute amplitude in that it showed significant differences with temporal frequency at low-moderate flash luminance with the corneal electrode, whereas the skin electrode B-PhNR ratio showed this to most flash strengths except the lowest luminance and one highest luminance value. These are illustrated in Fig. 3 and statistical significance according to flash luminance compiled in Table 1.

### PhNR Gaussian curve

**Fig. 4:**
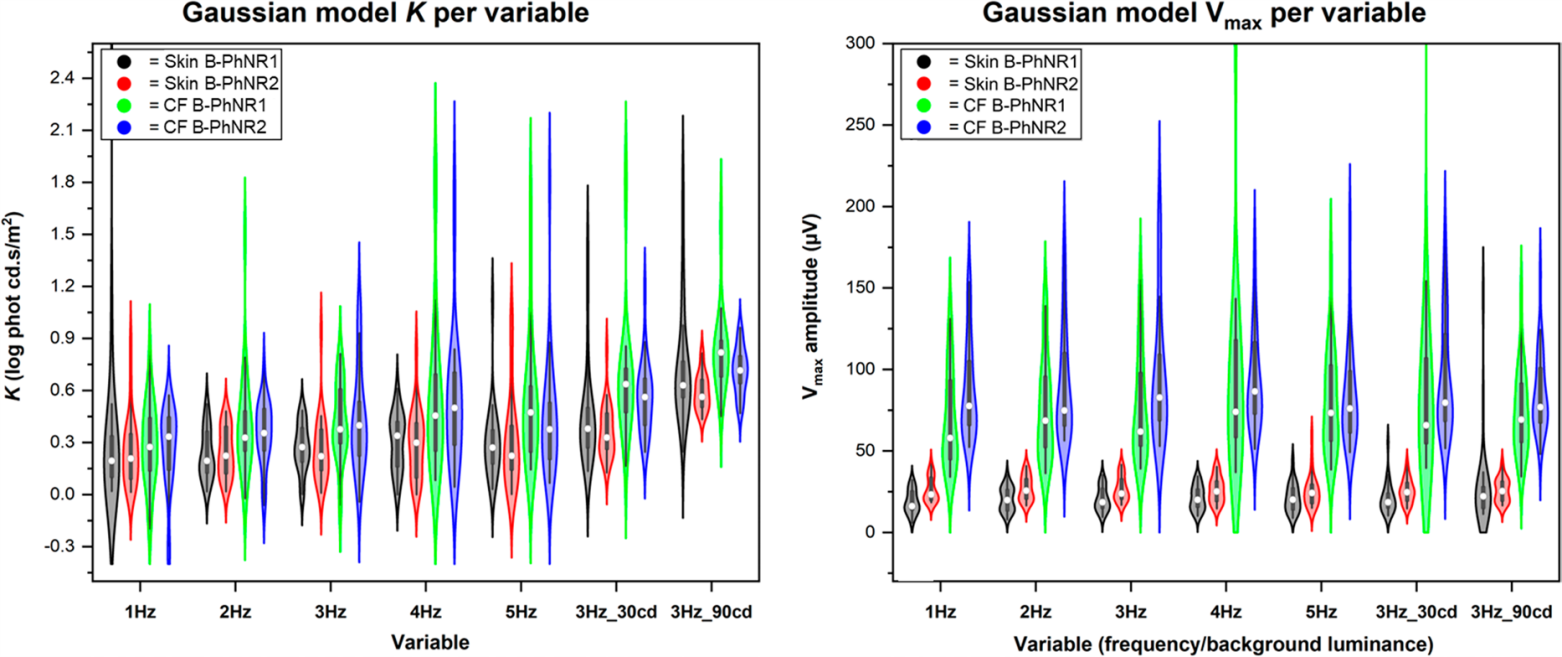
Violin plot illustrating the Gaussian model parameters *K* (left panel) and *V*_*max*_ (right panel) according to variable (i.e. temporal frequency or background luminance)(x-axis) and measurement type (black = skin B-PhNR1, red = skin B-PhNR2, green = corneal fibre B-PhNR1, blue = corneal fibre B-PhNR2). This is plotted for the *K* (left panel) and *V*_*max*_ (right panel). Violin plots illustrate the distribution of the data (coloured shape) where the shape indicates the approximate frequency of data points in each region. The white circle indicates the median, thick grey line indicates the interquartile rate, and thin line indicates 1.5 × the interquartile range. These data were plotted with exclusion of two outlying data points of the model export (PhNR006 LE data for corneal fibre B-PhNR2 *V*_*max*_ and PhNR10 RE data for corneal fibre B-PhNR1 *V*_*max*_ 3Hz30cd were excluded)

The overall effect size at individual luminance points appeared small with altered temporal frequency (Fig. 4), despite statistical significance at some individual luminance points. The effect of temporal frequency on the PhNR Gaussian model curve was then explored to account for all individual luminance points from the same subject. The derived *V*_*max*_ and *K* parameters showed no significant differences in PhNR1 or PhNR2 *V*_*max*_ or *K* across different temporal frequency with either electrode type (*p* > 0.05; Fig. 4). These are illustrated in Fig. 4.

### Background luminance

#### Absolute amplitude

**Fig. 5:**
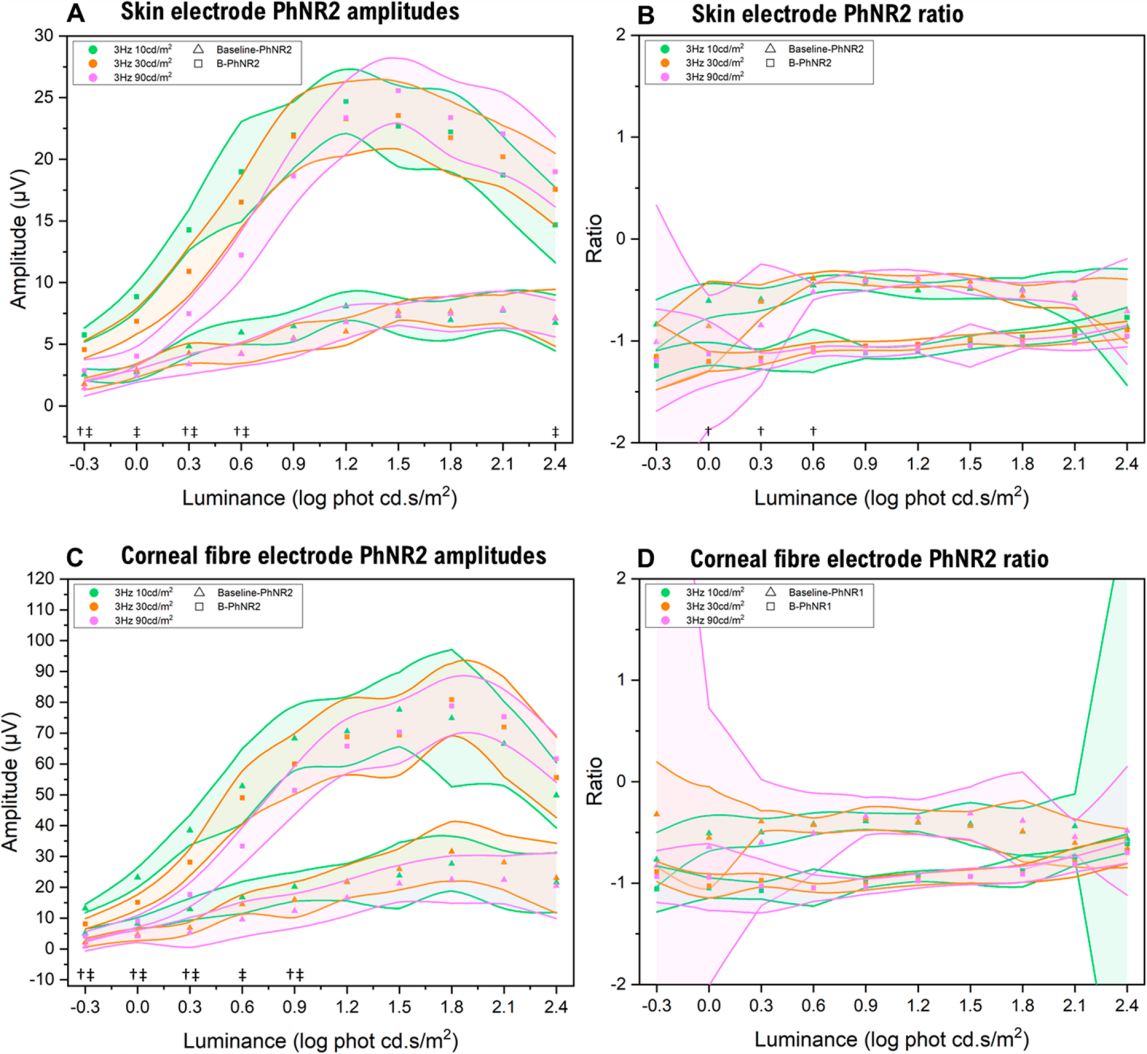
Effect of background luminance on the luminance-response function of the PhNR. For all panels, the median baseline-PhNR measurements are indicated by triangles, with B-PhNR measurements indicated by squares. The shaded areas around the median points are the 95% confidence intervals for each data point, with green indicating 10 cd/m^2^ background, orange indicating 30 cd/m^2^ background, and magenta indicating 90 cd/m^2^ background. Panel **A** shows the baseline-PhNR2 and B-PhNR2 amplitudes for the skin electrode at different background luminance. Panel **B** shows the baseline/PhNR2 ratio and B/PhNR2 ratio for the skin electrode at different background luminance. Panel **C** shows the baseline-PhNR2 and B-PhNR2 amplitudes for the corneal fibre electrode at different background luminance. Panel **D** shows the baseline/PhNR2 ratio and B/PhNR2 ratio for the corneal fibre electrode. Significant differences are indicated from † = significance observed for baseline-PhNR measurements at that flash luminance, and ‡ = statistical significance observed for B-PhNR measurements at that flash luminance

An increase in blue background luminance resulted in a smaller amplitude PhNR to low flash strengths for both electrode types (Fig. 5). This did not show any significant change in absolute amplitude to moderate-strong red flash stimuli (summarised in Table 2).

**Table 2:** Statistical significance (* = corrected *p*-value < 0.05) for PhNR measurement type with altering background luminance, according to each flash luminance strength

		PhNR differences with background luminance Flash luminance (log cd.s/m^2^)									
		– 0.3	0	0.3	0.6	0.9	1.2	1.5	1.8	2.1	2.4
Corneal fibre electrode	Baseline-PhNR amplitude	*	*	*	*	–	–	–	–	–	–
	B-PhNR amplitude	*	*	*	*	*	–	–	–	–	–
	Baseline-PhNR ratio	–	–	–	–	–	–	–	–	–	–
	B-PhNR ratio	–	–	–	–	–	–	–	–	–	–
Skin electrode	Baseline-PhNR amplitude	*	–	*	*	–	–	–	–	–	–
	B-PhNR amplitude	*	*	*	*	–	–	–	–	–	–
	Baseline-PhNR ratio	–	*	*	*	–	–	–	–	–	–
	B-PhNR ratio	–	–	–	–	–	–	–	–	–	–

#### Amplitude ratio

The PhNR ratio was less affected by background luminance, with no significant differences with background luminance for either electrode or measurement type, apart from the baseline-PhNR ratio to some dim flash strengths (Table 2).

The PhNR1 showed similar but fewer significant differences across the luminance-response series with background luminance, which are illustrated in Supplemental Fig. 3.

### PhNR gaussian curve

No significant differences were observed in PhNR1 or PhNR2 *V*_*max*_ with altered background luminance with either electrode type (*p* > 0.05; Fig. 4). In contrast, significant differences were observed for all *K* values with increased background luminance (*p* < 0.001), which corroborated the comparisons at each flash luminance, suggesting that background luminance increase only affects weaker flash strength PhNR amplitudes only.

### Effects of stimulus characteristics on other ERG parameters

To determine whether PhNR measures were influenced by earlier retinal generators of the ERG signal, a- and b-wave amplitudes were also assessed for differences with temporal frequency and background luminance.

#### Temporal frequency

The a-wave did not show any significant differences between temporal frequencies at any flash luminance for either electrode type (*p* > 0.05). The b-wave did not show any significant alterations with temporal frequency recorded with the corneal electrode (*p* > 0.05), which was similar for the skin electrode except for the two highest flash luminance (2.1 and 2.4 log cd.s/m^2^; *p* = 0.017, 0.012 respectively) comparing 1 and 4 Hz, and 1 and 5 Hz only. There was tendency for larger skin ERG a- and b-wave amplitudes with faster temporal frequency.

#### Background luminance

Significant differences were observed in the corneal electrode a-wave amplitude at low-moderate flash strengths (−0.3, 0, 0.3, 0.6, 0.9 and 1.2 log cd.s/m^2^; *p* < 0.05), as a-wave amplitudes tended to be smaller with greater background luminance. This was less marked for the skin electrode a-wave amplitude, where significant differences were only observed at the lowest (− 0.3 log cd.s/m^2^; *p* = 0.002) and one moderate flash strength (1.2 log cd.s/m^2^; *p* = 0.012). These changes are illustrated in Supplementary Fig. 4.

The b-wave amplitudes changed more markedly with increased background luminance, for the corneal electrode demonstrating significant differences at low-moderate flash strengths (− 0.3, 0, 0.3, 0.6 and 0.9 log cd.s/m^2^; *p* < 0.05). Similar findings were observed in the skin electrode with highly significant differences observed at low flash strengths (− 0.3, 0, 0.3 and 0.6 log cd.s/m^2^; *p* < 0.05). These b-wave curves (Supplementary Fig. 4) showed a general reduction at lower flash strengths with higher background luminance, but achieving similar maximal amplitude to strong flash strengths.

### Variability of the PhNR

#### PhNR variance changes with temporal frequency and luminance

**Fig. 6:**
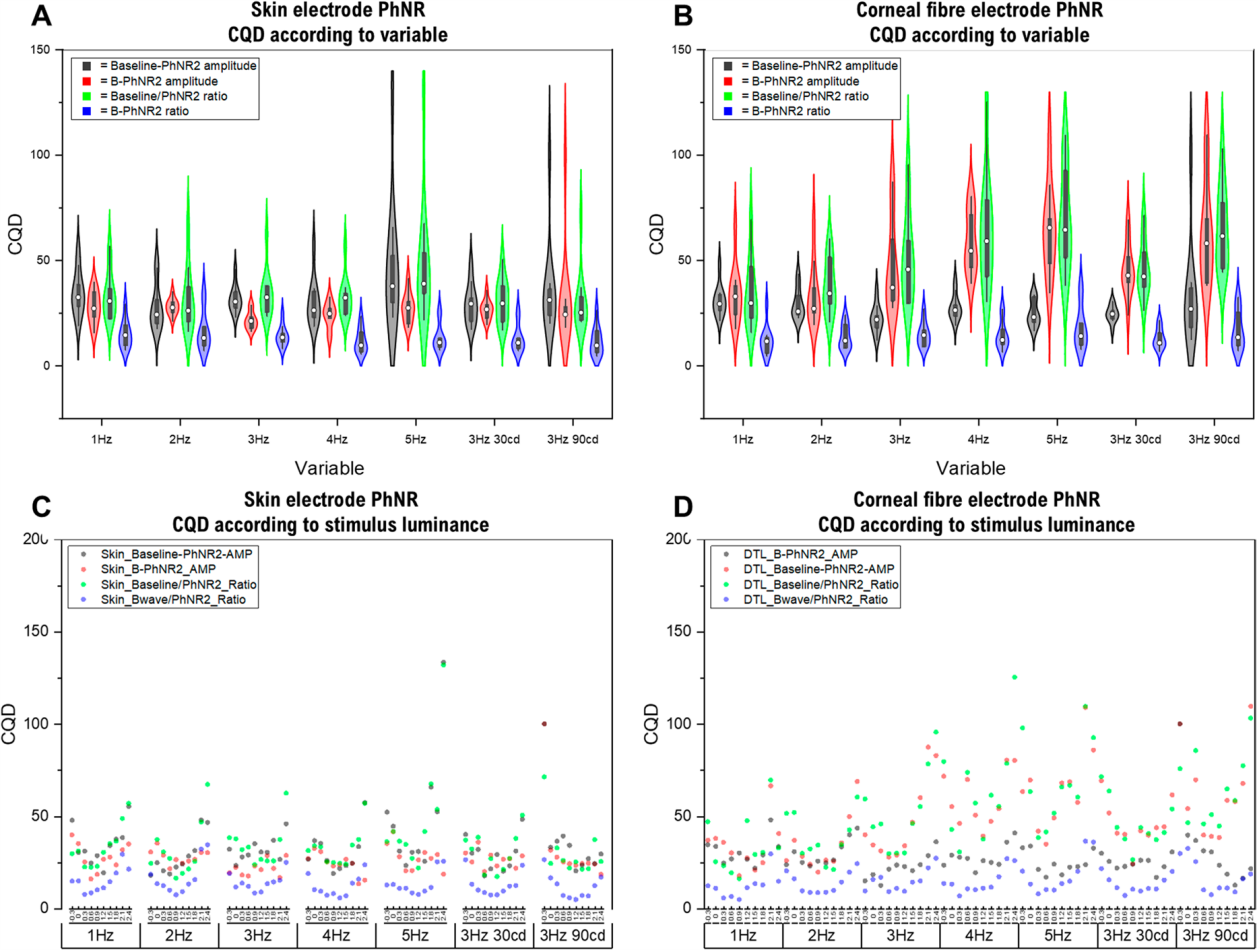
Coefficient of quartile deviation (CQD) variance measurements for different PhNR stimulus and measurement approaches. Panels **A** and **B** are violin plots of the grouped CQD for the PhNR with at different temporal frequency and background luminance variables. The effect is shown according to different measurement and/or electrode types (skin electrode panel A, corneal fibre electrode panel B). The CQD is demonstrated on the panel Y-axis, with variables of temporal frequency or background luminance shown on the X axis. The lower the CQD toward zero indicates a lower variance. The different violin plot colours indicate different measurement type as shown in the key. Panels **C**–**D** demonstrate the CQD at each flash luminance (log cd.s/m^2^) for the skin electrode (panel C) and corneal fibre electrode (panel D), with lowest luminance (− 0.3) on the left and increasing to the highest (2.4) on the right in 0.3 log unit steps. Each different icon represents a different PhNR measurement type (shown in the key)

Temporal frequency had a less effect on PhNR variance using a skin electrode compared to the corneal fibre electrode, evidenced by lower CQD. There were no significant differences in CQD across any baseline- or b-wave derived PhNR measurements with altered temporal frequency using the skin electrode (*p* > 0.05; all measurements), though qualitatively these appeared largest when using baseline measurements at 5 Hz temporal frequency (Fig. 6a, b). The corneal fibre PhNR variance showed significant differences in the B-PhNR2 amplitude between 1 and 3 Hz (*p* = 0.039), but with no significant difference observed for any B/PhNR2 ratio data (*p* = 0.585). In contrast, CQD were much higher for corneal fibre baseline-PhNR, baseline-PhNR ratio and B-PhNR ratio than the skin electrode; these were much more variable with higher frequency stimulation (Fig. 6a, b).

CQD was lowest when background luminance was lowest (10 cd/m^2^), and substantially increased for the 90 cd/m^2^ background luminance, with the corneal fibre electrode again having higher CQD than the skin electrode.

Stimulus luminance was also explored, by calculating individual CQD at each flash luminance at each variable for each electrode (Fig. 6c, d). Overall, these tended to illustrate more variance i.e. larger CQD at high and low flash luminance extremes, affecting the corneal fibre electrode more than the skin electrode. During recording, low luminance was associated with a lower signal–noise ratio, but at high luminance the variance could be attributed to blink artefacts affecting the PhNR. The skin electrode overall showed the least variance across the luminance-response series regardless of measurement type from baseline or b-wave, which appeared to have similar CQD regardless of background luminance and temporal frequency (Fig. 6c, d).

#### Differences in electrode type on the PhNR

The differences in luminance-response characteristics between electrode types were explored at two temporal frequencies; 1 Hz (the frequency suggested in the ISCEV extended protocol) and 4 Hz (selected by the authors as an ideal balance between data quality and recording speed).

Unsurprisingly, absolute *V*_*max*_ differed between corneal fibre and skin electrodes. The PhNR2 *V*_*max*_ from the skin electrode was ~ 30% the amplitude of the corneal fibre electrode for both 1 Hz (median 30.09, IQR 7.18) and 4 Hz (median 28.02, IQR 14.57) flash rates. Unexpectedly there was a significant difference in the flash luminance at which the peak amplitude (*V*_*max*_) occurred (e.g. Fig. 3a vs. 3c), described by the *µ* coefficient. For the corneal fibre B-PhNR2 at 1 Hz the *V*_*max*_ peak occurred at a median *µ* = 1.38 (IQR 0.33) log cd.s/m^2^ and for 4 Hz *µ* = 1.44 (IQR 0.49) log cd.s/m^2^, compared to the skin electrode 1 Hz median *µ* = 1.22 (IQR 0.29) log cd.s/m^2^ and 4 Hz *µ* = 1.32 (IQR 0.43) log cd.s/m^2^. There was therefore a shift of *µ* (reflecting the luminance at which *V*_*max*_ occurs) toward lower luminance values for the skin electrode by around 0.16 and 0.12 log cd.s/m^2^ for 1 Hz and 4 Hz, respectively. Similarly, sensitivity *K* shifted to lower luminances for the skin compared to corneal fibre electrode *K* = 0.12 (IQR 0.09) at 1 Hz and *K* = 0.15 (IQR 0.20) at 4 Hz. The lower *µ* [*lower luminance required to reach V*_*max*_] for the skin electrode consequently provided a larger descent following *V*_*max*_ that described a better Gaussian shape compared to the corneal electrode (e.g. Fig. 3a vs. 3c).

## Discussion

This study has evaluated the effects of temporal frequency, background luminance, electrode type, measurement approach and modelling of the luminance-response series of the PhNR. This is the first study to comprehensively evaluate all of these stimulus and recording parameters and to explore the potential inter-relationships between these common considerations in electrophysiological recordings.

The effect of increased temporal frequency had a small but significant impact of decreasing PhNR amplitudes when examining individual flash luminance values. However, when entire individuals luminance-response series were visualised, the significance of these differences reflected in the modelled Gaussian curve was insignificant and certainly the raw luminance-response curves appeared to show little change (Fig. 2). These data suggest that increased temporal frequency has little effect on the PhNR and its inner neural generators. A decrease in PhNR amplitude has been reported with increased temporal frequency when testing with one luminance value [26], but elsewhere no significant differences have been observed when considering the luminance-response series [16].

As these findings indicate that the PhNR luminance-response function is unaffected by temporal frequency between 1 and 5 Hz, variance between temporal frequencies was also explored. No significant differences were observed with altered temporal frequency in the variance, reflected by CQD, when increasing temporal frequency using a skin electrode. A slight increase in variance was observed when using a corneal fibre electrode with PhNR measured from the baseline, but not from the b-wave. Whilst one previous study reported a significant increase in variance at low flash frequencies using a single flash luminance [26], the present findings suggest this is not reflected in the PhNR luminance-response function. As the luminance-response series provides more detailed characterisation of PhNR characteristics, we would expect any physiological changes with temporal frequency to be exaggerated, which was not observed. It is the authors’ experience that slow flash frequencies lead to increased blinking and eye movement, with individuals becoming less rhythmically entrained to the stimulus and thus result in increased noise which may underly these observations, as observed by Hui et al. [26]. The current ISCEV PhNR extended protocol suggests that temporal frequency should not exceed 1 Hz [7], but does not cite empirical data and indeed the protocol includes the possibility of faster frequencies such as 4 Hz. Faster Frequency reduced recording duration; recording 100 sweeps per average and repeating, excluding rejections, resulted in a recording duration for the typical 1 Hz stimulus as 200 s (3 min 20 s), compared to the duration with a 4 Hz stimulus of only 50 s (< 1 min), quartering the time taken to record a PhNR. Increasing temporal frequency is of critical importance for clinical practice, particularly in challenging patient groups. The findings of this study suggest 4 Hz stimulation does not compromise the PhNR luminance-response dynamics or variance.

An increase in background blue luminance resulted in a significant decrease in PhNR amplitude to weaker flash strengths but had little effect to strong flash strengths, reflected as significant differences in the modelled Gaussian semi-saturation constant (*K*) but not maximal amplitude (*V*_*max*_)_._ This finding is consistent with previous reports that describe a decrease in the relative sensitivity of the light adapted flash ERG and PhNR with increased background luminance [25], and this has also been demonstrated at an extracellular level to be due to reduced sensitivity from cellular adaptation, pigment bleaching and response suppression [27]. Clinically, a stronger background may better silence the contribution of rods or decrease sensitivity of cones, and this work supports that the PhNR sensitivity is also reduced (shown by increased *K*). These changes with background luminance may theoretically decrease diagnostic utility in disease, and therefore maintaining a background luminance of 10 cd/m^2^ as suggested in the ISCEV extended protocol appears to be the optimal method [7].

This study shows that the PhNR measured from the b-wave (such as B-PhNR amplitude or B/PhNR ratio) shows the lowest variability, comparable to that of the a- and b-waves of the ffERG [28–30]. The PhNR recorded with skin electrodes has been shown to have lower inter-test variability than corneal fibre electrodes [31], although some other studies show higher variance in skin than corneal electrodes [16, 28]. We observed, using the widest range of PhNR flash strengths compared to previous studies, that there is increased variance at strong flash strengths, as noted in other studies [16, 29]. This was most notable with the corneal fibre electrode at low- and high flash luminance, whereas the skin electrode had more consistent variance across the luminance-response series (Fig. 6). The mechanisms behind this are intriguing, but may reflect the larger confounding effects of blink or eye movements with corneal fibre electrodes compared with skin electrodes, as the former will introduce a movement of the electrode itself whereas skin electrodes remain relatively static with such movements. Our data support that the skin electrode overall shows the lowest variance when recording the PhNR, which we believe reflects less confounding effects of blink and eye movement potentials from its infraorbital electrode position.

*Generally*, the major negativity reflected PhNR2 and was easier to measure in most participants. Whilst PhNR1 has been used in a small number of studies [5, 21–23], the seminal work of the PhNR describing its physiological mechanisms used the major negativity following the i-wave, PhNR2 [1]. It is worth noting that to strong flash strengths, later ‘slow’ oscillations were observed in some participants, which occasionally made PhNR2 measurement challenging. Whilst the first positivity following the i-wave has been notionally termed the i_2_-wave [24], this and the later oscillations observed here certainly warrant further investigation as to their exact retinal origin. Combined with the work presented here, the accumulating evidence suggests that when using a red-on-blue flash the PhNR2 is less variable. Whilst the origin of PhNR1 is likely of similar, if not the same, physiological origin as PhNR2, the latter can be more reliably measured than the PhNR1 due to variable i-wave amplitude. Similarly, the PhNR1 has been less well applied in studies of RGC function, overall suggesting future studies may focus on the PhNR2 for consistency.

The luminance response series in our study required a significantly larger luminance to reach *V*_*max*_*,* alongside *K*, than reported previously. Previous studies have reported a wide range of flash luminances at which *V*_*max*_ of the PhNR occurs, in log cd.s/m^2^ such as − 0.10–0.2 [calculated using reported 9 mm pupil size] [13], 0.2 [14], 0.2 [17], 0.09–0.5 [calculated using reported 8 mm pupil size] [15], 0.40 [19], 0.51 [16], and 0.7 [32]. Therefore, whilst other studies highlight this large variation, our finding of *V*_*max*_ occurring above 1.22 log cd.s/m^2^ was quite atypical. It is likely this change reflects retinal illuminance as the *V*_*max*_ for the ERG b-wave in this study also occurred around 1.2–1.8 log cd.s/m^2^, which is higher than that reported elsewhere [9, 10]. We speculate that this effect may be due to natural pupils used in this study, which may decrease the relative retinal illuminance of stimuli, as has been demonstrated in the photopic luminance-response series of the ERG [33]. We do not believe that the 90 degree^2^ field size would have had a significant effect compared with a full-field (120 degree^2^) physiologically, as over ~ 50% of RGCs are topographically located within the central 16 degree field [34–37]. Certainly, for those wishing to employ these protocols in dilated eyes, further study may be needed to confirm the typical luminance at which *V*_*max*_ occurs.

The most interesting and unexpected finding from the data presented here is the appreciable difference in the luminance required to reach *V*_*max*_ between electrode types; PhNR *V*_*max*_ peaked at lower flash strengths using the skin electrode. The flash luminance producing *V*_*max*_ with the corneal fibre electrode was 1.30–1.38 log cd.s/m^2^ compared to 1.15–1.30 log cd.s/m^2^ (1 Hz & 4 Hz, respectively) with the skin electrode. This showed 0.12–0.16 log cd.s/m^2^ decrease for the skin electrode compared to the corneal fibre electrode, was similar for *K* of 0.12–0.15 log cd.s/m^2^_._ This finding is particularly unusual and has not been reported previously when comparing electrode types across the PhNR luminance-response series [16]. Whilst a difference in *K* was reported between corneal and skin electrodes in association with the dark adapted ERG modelled with the Naka-Rushton function, no difference in the luminance at which *V*_*max*_ occurred was seen [38]. Given that PhNRs were recorded simultaneously with both electrodes, using the same filter settings and sampling rate, there is not a clear explanation for this finding. It is possible that factors such as volume conduction/filtering through the skin and tissues, orientation of the retinal generators relative to the skin versus corneal fibre electrode or perhaps the difference in spectral weighting or signal–noise ratios may each contribute to this difference. Further work may elaborate if this is a feature specific to the spectral stimulus combination, or may be more widely seen with broadband white flash stimuli or under scotopic conditions of the ffERG.

A Gaussian model was able to characterise more of the luminance-response function of the PhNR, compared with previous studies using the Naka-Rushton function. The descending limb of the PhNR ‘hill’ has been proposed to be a consequence of the characteristic ‘photopic-hill’ of the b-wave, due to decreased amplitude of on-bipolar cell activity and delay in off-bipolar cell activity [10, 17]. Certainly, the i-wave, which is closely before the PhNR, also exhibits a Gaussian curve with increasing flash luminance [39]. We observed a steeper decrease in PhNR amplitude at strong flash strengths compared to the b-wave amplitude, suggesting that alterations in the b-wave do not entirely explain the PhNR ‘hill’. It could be hypothesised that at strong flash strengths, increased inhibitory or antagonistic inner retinal activity may suppress an excessive luminance signal to RGCs [40], which could cause a stronger decline in PhNR amplitude. Certainly, further research in this area may be able to elaborate this finding by studying the luminance-response function to long duration flashes that separate on- and off- pathway activity.

There are several limitations to the current work. Whilst we opted to test healthy participants to understand how PhNR stimulus and recording parameters behave, we can not truly translate how this may be altered in diseases where RGCs are dysfunctional. Furthermore, we opted to test with natural pupils to mimic clinical protocols, but this may introduce difference in the luminance-response function of the PhNR compared with dilated examinations. These factors should be considered for any laboratories considering these techniques, and individual laboratory reference data should be collected for clinical use.

## Conclusion

This study is the first to show that the optimal PhNR recording is to use natural pupils, skin electrodes and flash stimuli presented at 4p/sec to produce luminance-response curves with the lowest variance, best model fit and most reliable PhNR measurements from the baseline as well as b-wave. For maximal diagnostic sensitivity background luminance above 10 cd/m^2^ should be avoided. Caution should be practiced when comparing between electrode types due to differences in the luminance where *V*_*max*_ occurs between electrodes.

## Supplementary Information

Below is the link to the electronic supplementary material.

Supplementary file1 (DOCX 1935 KB)
