# Supplementary material for: The luminance-response function of the photopic negative response (PhNR): analysing different stimulation, recording and measurement approaches
Source: Doc Ophthalmol. 2026 Apr 7;152(3):341–56. doi: 10.1007/s10633-026-10102-0 (PMC13194254; doi:10.1007/s10633-026-10102-0)
Supplement: Supplementary file 1 — Supplementary file1 (DOCX 1935 KB) [file 10633_2026_10102_MOESM1_ESM.docx]

# Supplemental Table 1 – Root mean square error (RMSE) of gaussian fit

| **Gaussian model fitting B-PhNR** | | | | | |
| --- | --- | --- | --- | --- | --- |
| **Variable & component** | | **% of series where 2 data points removed** | **% of series where 1 data points removed** | **% of series where 0 data points removed** | **Resulting Gaussian fit RMSE (median ± IQR)** |
| 1Hz | CF B-PhNR1 | 8.33 | 41.67 | 50.00 | 3.51 (1.87-5.04) |
| 2Hz |  | 29.17 | 20.83 | 50.00 | 2.29 (1.31-3.60) |
| 3Hz |  | 0.00 | 33.33 | 66.67 | 2.35 (1.82-4.12) |
| 4Hz |  | 0.00 | 37.50 | 62.50 | 2.64 (1.81-3.64) |
| 5Hz |  | 4.17 | 33.33 | 62.50 | 2.38 (1.64-3.29) |
| 3Hz 30cd/m^2^ |  | 16.67 | 29.17 | 54.17 | 2.54 (2.30-4.37) |
| 3Hz 90cd/m^2^ |  | 0.00 | 16.67 | 83.33 | 2.87 (2.08-3.59) |
| 1Hz | CF B-PhNR2 | 4.17 | 16.67 | 79.17 | 3.37 (2.49-4.65) |
| 2Hz |  | 0.00 | 41.67 | 58.33 | 2.83 (1.85-4.13) |
| 3Hz |  | 0.00 | 16.67 | 83.33 | 3.42 (2.61-5.68) |
| 4Hz |  | 8.33 | 16.67 | 75.00 | 2.73 (2.06-4.96) |
| 5Hz |  | 20.83 | 12.50 | 66.67 | 2.86 (2.19-3.50) |
| 3Hz 30cd/m^2^ |  | 8.33 | 16.67 | 75.00 | 3.24 (2.32-4.42) |
| 3Hz 90cd/m^2^ |  | 8.33 | 12.50 | 79.17 | 3.11 (1.80-4.01) |
| 1Hz | Skin B-PhNR1 | 16.67 | 16.67 | 66.67 | 1.29 (0.82-1.95) |
| 2Hz |  | 12.50 | 29.17 | 58.33 | 0.87 (0.60-1.17) |
| 3Hz |  | 20.83 | 29.17 | 50.00 | 0.81 (0.50-1.15) |
| 4Hz |  | 25.00 | 25.00 | 50.00 | 0.76 (0.50-1.08) |
| 5Hz |  | 25.00 | 33.33 | 41.67 | 0.68 (0.56-0.92) |
| 3Hz 30cd/m^2^ |  | 33.33 | 25.00 | 41.67 | 0.85 (0.58-1.15) |
| 3Hz 90cd/m^2^ |  | 16.67 | 20.83 | 62.50 | 0.99 (0.77-1.52) |
| 1Hz | Skin B-PhNR2 | 12.50 | 33.33 | 54.17 | 1.29 (0.78-2.10) |
| 2Hz |  | 16.67 | 25.00 | 58.33 | 0.74 (0.47-1.17) |
| 3Hz |  | 16.67 | 41.67 | 41.67 | 1.03 (0.76-1.33) |
| 4Hz |  | 12.50 | 37.50 | 50.00 | 0.88 (0.63-1.19) |
| 5Hz |  | 16.67 | 8.33 | 75.00 | 0.96 (0.60-1.37) |
| 3Hz 30cd/m^2^ |  | 25.00 | 16.67 | 58.33 | 0.89 (0.72-1.35) |
| 3Hz 90cd/m^2^ |  | 29.17 | 20.83 | 50.00 | 1.04 (0.71-1.52) |

***Supplementary Table 1 - Gaussian model fitting summary for B-PhNR1 and B-PhNR2 data.*** *The protocol and variable (temporal frequency or background luminance) is shown in the left column, followed in the next column by the electrode type (skin or corneal fibre) and PhNR component measured. The following columns summarise the percentage of data for each protocol which had 2, 1 or 0 data points removed to optimise the Gaussian fit. The resultant RMSE are provided in the last column (as median and interquartile range).*

# Supplemental Figure 1 Example Gaussian curve fitting and main parameters from the model.


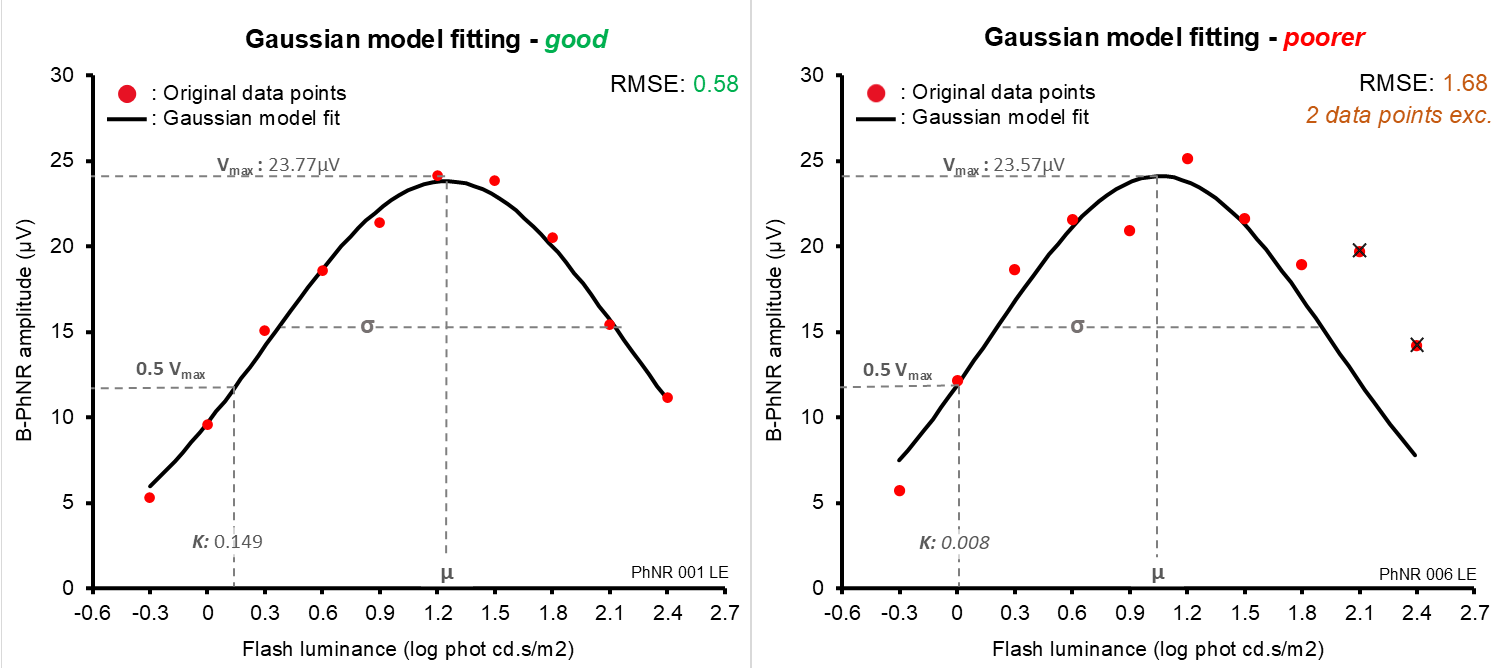


***Supplemental Figure 1 – Illustration of the gaussian model fit to the PhNR luminance response series****. Data provided are those from B-PhNR2 for the skin electrode at 3Hz temporal frequency, for participant PhNR 001 (left eye) in the left panel, and PhNR 006 (left eye) in the right panel. The panels show the original data points (red circles) with the gaussian model fit (black line) plotted by B-PhNR amplitude (y-axis) by log flash luminance (x-axis). Model coefficients of Vmax and K are indicated in grey. The left panel shows a good model fit providing a RMSE of 0.58. The right panel shows a poorer model fit whereby two data points were excluded to improve RMSE (crossed data points).*

# Supplemental Figure 2

Effects of temporal frequency on PhNR1.


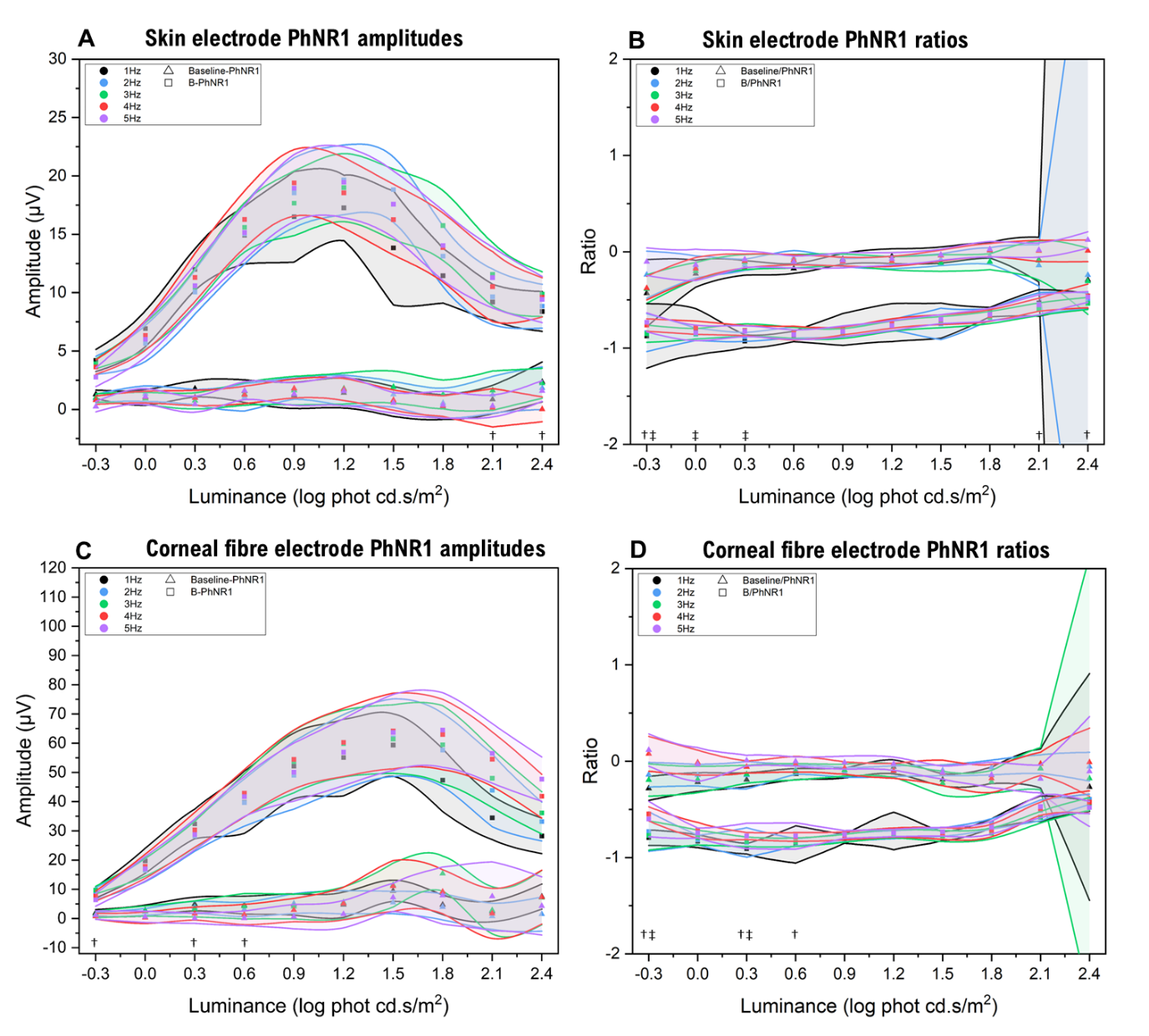


**Supplemental Figure 2 - Effect of temporal frequency on the luminance-response function of the PhNR1**. For all panels, the median baseline-PhNR measurements are indicated by triangles, with B-PhNR measurements indicated by squares. The shaded areas around the median points are the 95% confidence intervals for each data point. Panel A shows the absolute baseline-PhNR2 and B-PhNR2 amplitudes for the skin electrode across different temporal frequencies (1-5Hz). Panel B shows the baseline/PhNR2 ratio and B/PhNR2 ratio for the skin electrode across different temporal frequencies. Panel C shows the absolute baseline-PhNR2 and B-PhNR2 amplitudes for the corneal fibre electrode across different temporal frequencies (1-5Hz). Panel D shows the baseline/PhNR2 ratio and B/PhNR2 ratio for the corneal fibre electrode across different temporal frequencies. Significant differences are indicated by †= significance observed for baseline-PhNR measurements at that flash luminance, and ‡= statistical significance observed for B-PhNR measurements at that flash luminance.

# Supplemental Figure 3

Effects of background luminance on PhNR1.


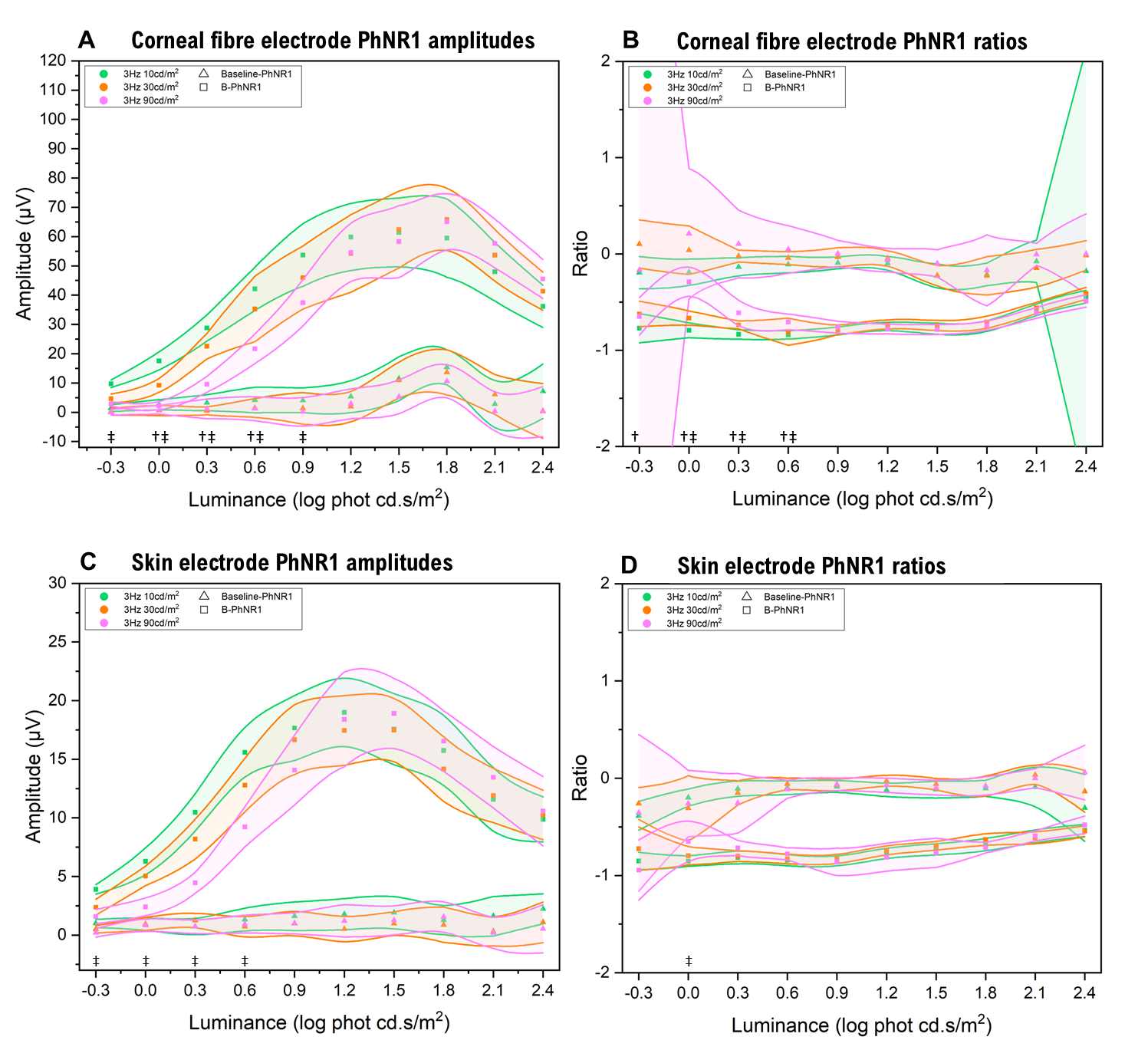


**Supplemental Figure 3 - Effect of background luminance on the luminance-response function of the PhNR1**. For all panels, the median baseline-PhNR measurements are indicated by triangles, with B-PhNR measurements indicated by squares. The shaded areas around the median points are the 95% confidence intervals for each data point. Panel A shows the absolute baseline-PhNR1 and B-PhNR1 amplitudes for the corneal fibre electrode across different background luminance (10cd/m^2^ green, 30cd/m^2^ orange, 90cd/m^2^ magenta). Panel B shows the baseline/PhNR2 ratio and B/PhNR2 ratio for the corneal fibre electrode across different background luminance frequencies. Panel C and D mirror the same as panels A and B for the skin electrode. Significant differences are indicated by †= significance observed for baseline-PhNR measurements at that flash luminance, and ‡= statistical significance observed for B-PhNR measurements at that flash luminance.

# Supplemental Figure 4

Effects of temporal frequency and background luminance on other ERG components


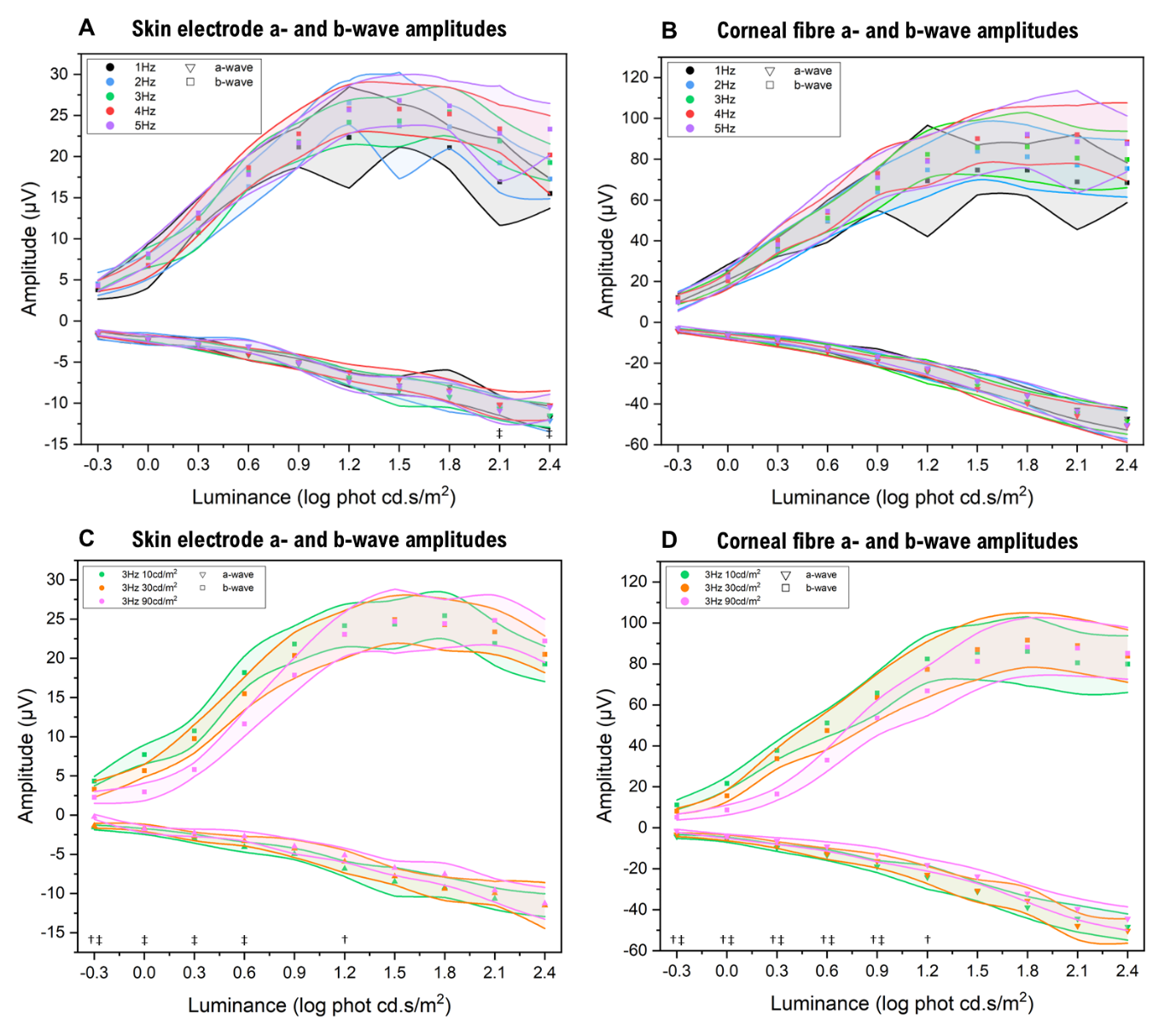


**Supplemental Figure 4 - *Photopic ERG luminance-response series a- and b-waves with differing temporal frequency (A, B) and background luminance (C, D).*** *Corneal fibre electrode data are displayed in the panel B and D and skin electrode data displayed in panel A and C. For all panels, the median a-wave amplitude is shown by the downward triangle, with median b-wave amplitude shown by squares. The shaded areas around the median points are the 95% confidence intervals for each data point. Each temporal frequency corresponds to a respective colour (1 Hz black, 2 Hz blue, 3 Hz green, 4 Hz red, 5 Hz purple), as do background luminance (3 Hz 10cd/m^2^ green, 3 Hz 30cd/m^2^ orange, 3 Hz 90cd/m^2^ pink). Significant differences are indicated from †= significance observed for a-wave measurements at that flash luminance, and ‡= statistical significance observed for a-b-wave measurements at that flash luminance*
